# Supplementary material for: Causal Relationship between Aging and Anorexia Nervosa: A White-Matter-Microstructure-Mediated Mendelian Randomization Analysis
Source: Biomedicines. 2024 Aug 16;12(8):1874. doi: 10.3390/biomedicines12081874 (PMC11351342; doi:10.3390/biomedicines12081874)
Supplement: Supplementary file 1 [file biomedicines-12-01874-s001.zip › Supplementary Figure/Figure S4/Singe SNP plot of MR analysis for TL to ubm-b-1730.pdf]

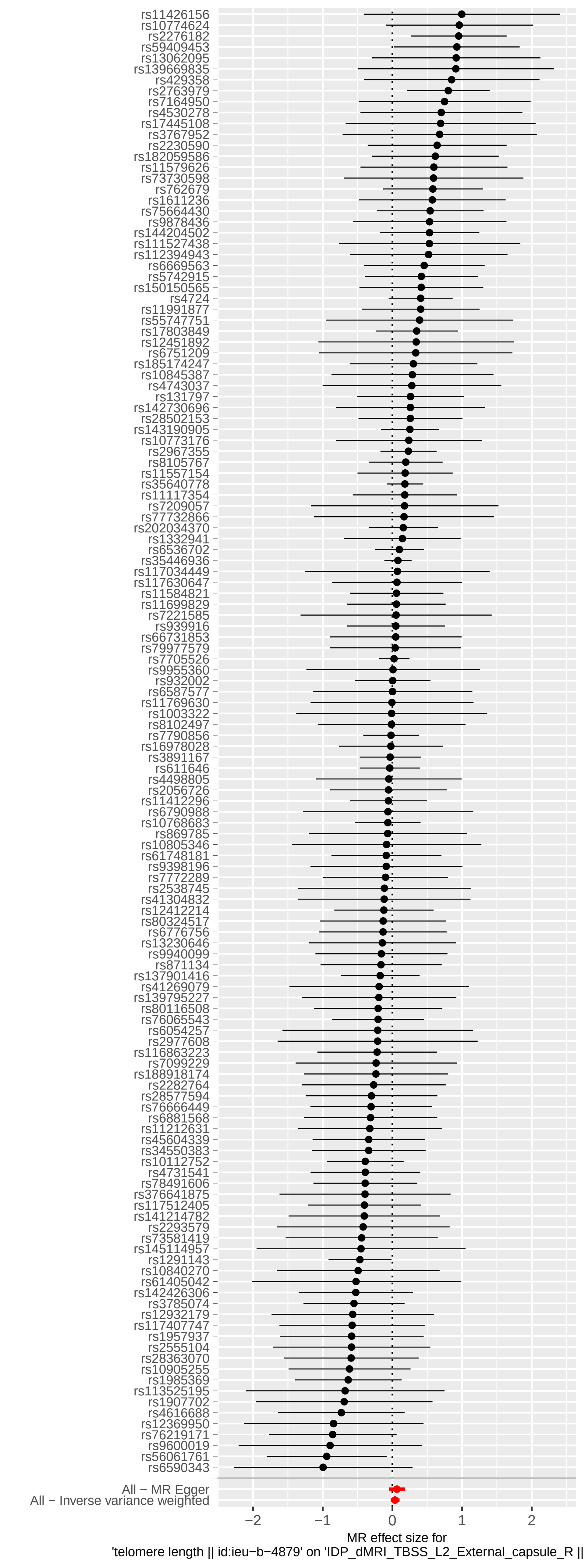
rs11426156
rs10774624
rs2276182
rs59409453
rs13062095
rs139669835
rs429358
rs2763979
rs7164950
rs4530278
rs17445108
rs3767952
rs2230590
rs182059586
rs11579626
rs73730598
rs762679
rs1611236
rs75664430
rs9878436
rs144204502
rs111527438
rs112394943
rs6669563
rs5742915
rs150150565
rs4724
rs11991877
rs55747751
rs17803849
rs12451892
rs6751209
rs185174247
rs10845387
rs4743037
rs131797
rs142730696
rs28502153
rs143190905
rs10773176
rs2967355
rs8105767
rs11557154
rs35640778
rs11117354
rs7209057
rs77732866
rs202034370
rs1332941
rs6536702
rs35446936
rs117034449
rs117630647
rs11584821
rs11699829
rs7221585
rs939916
rs66731853
rs79977579
rs7705526
rs9955360
rs932002
rs6587577
rs11769630
rs1003322
rs8102497
rs7790856
rs16978028
rs3891167
rs611646
rs4498805
rs2056726
rs11412296
rs6790988
rs10768683
rs869785
rs10805346
rs61748181
rs9398196
rs7772289
rs2538745
rs41304832
rs12412214
rs80324517
rs6776756
rs13230646
rs9940099
rs871134
rs137901416
rs41269079
rs139795227
rs80116508
rs76065543
rs6054257
rs2977608
rs116863223
rs7099229
rs188918174
rs2282764
rs28577594
rs76666449
rs6881568
rs11212631
rs45604339
rs34550383
rs10112752
rs4731541
rs78491606
rs376641875
rs117512405
rs141214782
rs2293579
rs73581419
rs145114957
rs1291143
rs10840270
rs61405042
rs142426306
rs3785074
rs12932179
rs117407747
rs1957937
rs2555104
rs28363070
rs10905255
rs1985369
rs113525195
rs1907702
rs4616688
rs12369950
rs76219171
rs9600019
rs56061761
rs6590343
All − MR Egger
All − Inverse variance weighted
−2
−1
0
1
2
MR effect size for
'telomere length || id:ieu−b−4879' on 'IDP_dMRI_TBSS_L2_External_capsule_R ||
